# Classification of Textile Samples Using Data Fusion Combining Near- and Mid-Infrared Spectral Information

**DOI:** 10.3390/polym14153073

**Published:** 2022-07-29

**Authors:** Jordi-Roger Riba, Rosa Cantero, Rita Puig

**Affiliations:** 1 Electrical Engineering Department, Universitat Politècnica de Catalunya, Rambla Sant Nebridi 22, 08222 Terrassa, Spain; 2 Department of Computer Science and Industrial Engineering, Universitat de Lleida, Pla de la Massa 8, 08700 Igualada, Spain; rosa.cantero@udl.cat (R.C.); rita.puig@udl.cat (R.P.)

**Keywords:** textile waste, classification, data fusion, NIR spectroscopy, FTIR spectroscopy, MIR spectroscopy, circular economy, post-consumer waste

## Abstract

There is an urgent need to reuse and recycle textile fibers, since today, low recycling rates are achieved. Accurate classification methods for post-consumer textile waste are needed in the short term for a higher circularity in the textile and fashion industries. This paper compares different spectroscopic data from textile samples in order to correctly classify the textile samples. The accurate classification of textile waste results in higher recycling rates and a better quality of the recycled materials. The data fusion of near- and mid-infrared spectra is compared with single-spectrum information. The classification results show that data fusion is a better option, providing more accurate classification results, especially for difficult classification problems where the classes are wide and close to one another. The experimental results presented in this paper prove that the data fusion of near- and mid-infrared spectra is a good option for accurate textile-waste classification, since this approach allows the classification results to be significantly improved.

## 1. Introduction

The textile industry is currently one of the main economic activities worldwide. It amounts to approximately EUR 178 billion in Europe, and about 1.7 million jobs are generated throughout its value chain [1]. Textile is produced and consumed linearly, as low recycling rates are achieved nowadays. Specifically, fashion consumption has increased to an average of 26 kg of textile products per person each year [2]. However, the fashion and textile industries still significantly pollute because of the intensive consumption of energy, chemicals and water, with this fact being boosted by present “buy-and-throw-away” habits. These habits must be reversed to improve sustainability and to reduce the associated social and environmental impacts [3]. Circular economies aim at providing maximum usefulness and value of materials, components and products [4]. Circular economies can help to achieve these goals, since it allows product life to be extended via the reuse of textiles, while preventing the use of toxic components [5]. Textile reuse and recycling lessens environmental impacts compared with incineration and landfilling [6] while reducing textile-fiber production [7]. The fashion and textile industries should gradually be involved in a circular economy, since this approach allows the life of textile fibers to be extended and to be retained in a closed circuit, so that they can be reused [8]. To this end, production and consumption habits require to be modified to minimize the environmental impacts and preserve natural resources as much as possible [9,10]. Nowadays, many efforts are being applied for a deeper implementation of circular economies in different industrial activities, including the food and agricultural sectors [11], packaging [12] and textile production [13], among others.

European institutions are implementing strict environmental regulations in the textile sector. In fact, in 2018, a European regulation (Directive (EU) 2018/851) [14] was approved with a mandate to implement the selective collection of textile waste before 1 January 2025. This new legislation has the aim to increase the recycling rate of textile post-consumer waste. However, currently, textiles are habitually manually sorted. Manual sorting presents many disadvantages, such as low processing rates, high cost and not being compatible with a full automation as required for processing huge quantities of textile materials [15]. Therefore, it is necessary to implement an automatic classification system to allow the correct separation of the different textile fibers to be performed, thus increasing their recyclability and the added value of recycled textile materials. This approach, which is under research and development, would contribute to moving from the current linear textile system to a more circular one. This paper aims at contributing to the sensing and classifying stages as conducted by a machine for automatic textile-sorting by combining optical sensors and multivariate statistical algorithms.

Measurements based on optical methods are appealing because they avoid any contact with the sample [16]. Spectroscopic techniques, such as near- and mid-infrared spectroscopy (NIR and MIR), combined with chemometric modeling offer a potential solution to this textile-classification problem. They are fast, non-destructive, cost-effective and environmentally friendly methods. NIR spectroscopy has been successfully applied as an analytical technique in different areas, such as the food [17,18], agriculture [19,20], fuel [21] and paper industries [22], among others, due to several advantages, including fast response, being reagent-free and noninvasiveness. MIR has been applied to identify cellulose-based fibers [23], natural rubber samples [24], changes in engine-oil properties [25] and to determine the thermal stability of explosives [26], among many other applications. As spectroscopic methods gain more widespread use, the testing, characterization, calibration and validation of the measurements and modeling algorithms become essential [27]. NIR has already been reported as an interesting technology to identify different textile samples. For example, an exploratory study based on NIR spectroscopy for recognizing textile materials was carried out [28]. Another work used principal-component analysis–linear-discrimination analysis (PCA-LDA) and the soft independent modeling of class analogy (SIMCA) to identify three pure fibers and their mixtures in the NIR range [29], but they did not split the whole set of samples into calibration and prediction sets, so the work cannot be directly used for real-world applications. An online NIR library including seven pure fibers and six binary mixtures was created and identified by means of convolutional neural networks [30]. However, these works only deal with NIR spectral information.

Data-fusion methods are based on combining data acquired by multiple sensors to generate more accurate, useful and consistent information than that obtained with the use of a single sensor. Multi-sensor data also combine the advantages of using different sensors [31], because they allow on to expand the available information of the samples, thus enhancing model accuracy and avoiding some limitations associated with individual sensors [32]. The fusion of data obtained with complementary techniques was recently reported as a promising option to improve the completeness and accuracy of the results [33]. Data fusion was tested and reported in different areas, such as health [34,35], agriculture [36,37,38] and food [17,39], among others. In this paper, a multi-sensor data-fusion approach is applied to classify textile fibers based on NIR and MIR spectroscopy. However, NIR and MIR involve a large number of correlated features [20], so it is important to apply suitable feature extraction or reduction methods to enhance the discrimination accuracy [40].

Although several publications related to NIR spectroscopy, as a method to identify different textile fibers (cotton, wool, polyester, etc.) [41,42] and some blend mixtures [30], recently appeared and papers related to MIR spectroscopy can also be found (to measure textile degradation [43] and for textile-fiber identification [13]), to the best of our knowledge, there are no studies related to the data fusion of NIR and MIR spectra in textile industry yet.

Thus, the aim of this paper is to develop a sorting system able to separate the most common pure textile fibers (cotton, linen, wool, silk, polyester, polyamide and viscose), as well as binary mixtures of viscose–polyester and cotton–polyester using data fusion of NIR and MIR spectra. The classification system needs to be as accurate as possible to assure a high quality of the recycled materials. Therefore, data fusion of near and mid infrared spectra is used to obtain an accurate classification.

The mathematical treatment of the spectra consists of three steps. First, principal-components analysis (PCA) is applied, followed by the canonical-variate analysis (CVA) algorithm, and finally, the obtained data are classified by applying the *k*-nearest neighbor (*k*NN) classifier.

The paper is structured in seven sections, with the first being the Section 1. In Section 2, NIR, MIR and combined NIR + MIR spectroscopy are described, followed by the description of the textile samples (Section 3). In Section 4, the applied algorithms are presented. Next, Section 5 shows the NIR and MIR spectra of some samples, and finally, experimental results (Section 6) and conclusions (Section 7) are discussed.

## 2. NIR and MIR Spectroscopy

### 2.1. MIR Spectroscopy

The middle infrared (MIR) electromagnetic radiation (wavenumber from 4000 to 400 cm^−1^) is energetic enough to cause transitions between the rotational and vibrational levels of the molecular bonds. The absorption of this radiation is highly sensitive to the type of bonds in a certain molecule. This is why this region of the spectrum is widely used in both the qualitative and quantitative analyses of organic molecules and polymers.

MIR technology is often coupled with Attenuated Total Reflectance equipment (ATR) to avoid any pre-treatment of the solid and liquid samples. ATR equipment consists of an optic crystal (with a high refraction index) that receives the IR beam from one side, while the other side is in contact with the sample. Through the crystal, the sample receives an IR beam, absorbs energy in specific regions and reflects the rest to the crystal again. Several reflections can take place until the beam arrives to the detector.

The MIR spectra of the textile samples were acquired using a PerkinElmer spectrometer (Spectrum Two, S/N 114153; Shelton, CT, USA) equipped with an ATR module. The FTIR spectrometer provides spectral information within the wavenumber interval of 4000–450 cm^−1^, having 1 cm^−1^ resolution and averaging four scans, thus obtaining 3551 data points per sample.

### 2.2. NIR Spectroscopy

The near infrared region (NIR) has a range from 750 to 2500 nm, and the radiation absorbed is due to overtones and combinations from fundamental vibrations produced in the medium infrared (MIR). These bands provide useful information but have important limitations due to their low intensity, which is two or three times lower than those that appear in the medium infrared. Only absorption bands due to vibrations of high frequency are observed in the NIR spectra. In addition, in the NIR region, bands and overtones overlap, thus making it difficult to interpret the spectrum.

Hydrogen, being the lightest of the atoms, vibrates more widely during stretching vibrations. Consequently, almost all the absorption bands observed in the NIR come from overtones of the stretching vibrations of AH_x_ groups (mostly CH, OH and NH). There are few bands apart from those due to the overtones of CH, OH and NH groups, although in some cases, PH and SH bands can be observed.

The near infrared (NIR) spectra of the textile samples were acquired by using an FOSS spectrometer (XDS^TM^ OptiProbe Analyzer, FOSS AnalyticalA/S, Hillerod, Denmark) equipped with a fiber-optic probe to perform reflectance measurements. The instrument was controlled with Vision Software^TM^ (version 6.1, FOSS AnalyticalA/S, Hillerod, Denmark). The NIR spectrometer provides spectral information within the wavelength interval of 400–2499.5 nm with a resolution of 0.5 nm, using an average of 32 scans, thus providing a total of 4200 data points per sample. The shorter wavelengths correspond to the visible spectrum, with the NIR spectrum corresponding to the 1100–2500 nm wavelength interval. However, the signal near 2500 nm contains more noise, thus making the results less accurate. Therefore, it was decided to analyze the 1100–2200 nm interval, so each spectrum included a total of 2201 wavelengths.

### 2.3. Combined NIR + MIR Spectroscopy

As discussed above, molecular vibrations are the source of the absorption bands located in the MIR (middle infrared) region, while the absorption in the NIR (near infrared) region is due to the overtones and band combination of the previous fundamental vibrations that occur in the middle infrared.

Data fusion allows one to comprehensively combine original data from the MIR and NIR, taking advantage of the synergistic and complementary information provided by both techniques.

Therefore, studies based on the fusion of NIR and MIR spectra can provide more accurate and complete information about the samples under analysis. Data-fusion strategies are successfully implemented in different areas, improving the results obtained with the use of individual techniques, which provide only limited, partial information.

NIR + MIR spectroscopy refers to the combined use of NIR and MIR spectra for specific purposes, in the form of data fusion. This approach increases the amount of information of a given sample, which allows better classification results to be obtained. The specific case of merging MIR and NIR data is beginning to be applied in various fields, such as food technology [17], medical diagnosis [34], medicinal-herb origin [44,45], etc.

In this case, NIR and MIR are combined because some problems were found using NIR alone when dealing with dark-colored samples (due to the low reflectance of the radiation) and with wet samples (due to the wide and intense peak of the water, which overlaps other peaks in the NIR spectra). These problems may be solved using MIR spectra due to the complementarity of both techniques, although MIR is more difficult to apply on an industrial scale. However, MIR sensors are likely to evolve in the coming years to be more easily industrially applicable.

## 3. Sample Collection and Identification

This paper analyzes textile samples that were collected from the catalogs of several companies. These textile samples include natural fibers (cotton, linen, wool and silk samples), synthetic and artificial fibers (viscose, polyamide and polyester samples) and mixtures between both types. It is noted that artificial fibers come from the transformation of natural products (for example, viscose is obtained from cellulose), whereas synthetic fibers are made from polymers that are derived from petrochemicals.

The selection of textile fibers aimed to include maximum variability, so different presentations (fabric or yarn) and multitude of colors (from dark to light) were included in the analyzed set of samples. For an accurate identification, all textile fibers had a code and a description including the color, presentation type, manufacturer and catalog reference.

Samples were provided by different companies (a total of 8) and were from different years (from 2016 to 2019). A total of 52 different commercial catalogs were used to obtain individual samples for each of the three studies performed, which are described in Section 6. The number of catalogs used in each study was 25 catalogs for the first study, 11 for the second one and 25 for the third one. Samples could not be visually classified because their appearance widely varied, so the exact composition of the sample was provided by the companies. The composition of each sample was correct because it was exactly defined in the catalog provided by the company.

Spectral acquisition was conducted via the contact between the sensor and the sample. All samples were at least 9 cm^2^ in size, and the thickness widely varied because there were winter and summer pieces of textile. When the thickness was not sufficient, the sample was folded 2–3 times before spectral acquisition to obtain the desired thickness.

## 4. Applied Algorithms

**Figure 1:**
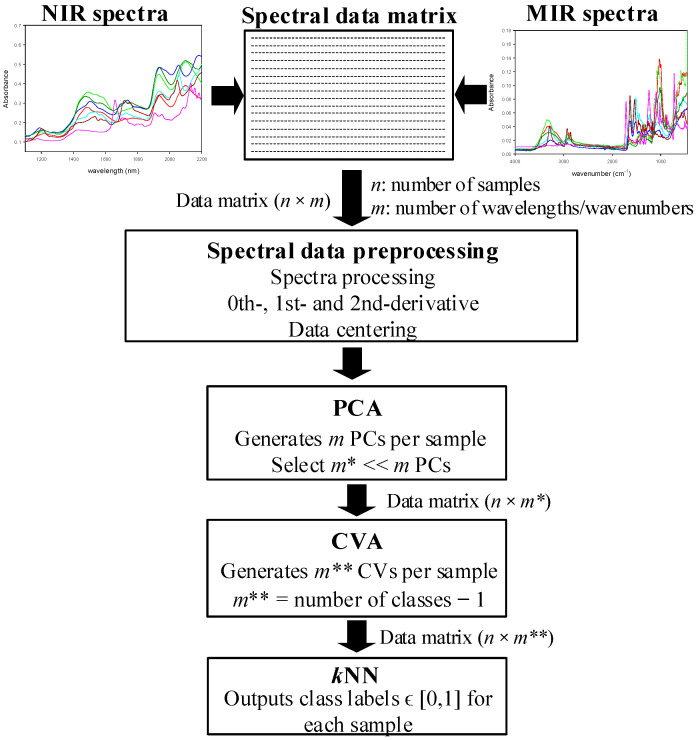
Diagram of classification strategy applied to spectral data for classifying unknown incoming fiber samples according to pre-established classes of textile fibers.

Figure 1 shows the mathematical strategy applied to classify unknown incoming textile fibers according to pre-established classes, where each class is defined by different fiber compositions. As shown in Figure 1, the raw spectral data were first preprocessed; next, the principal-component analysis (PCA) was applied, followed by the canonical-variate analysis (CVA) algorithm. Finally, the data were classified by applying the *k*-nearest neighbor (*k*NN) classifier.

### 4.1. Calibration and Prediction Data Subsets

To assess the accuracy of the analyzed mathematical classification algorithms, it is a common practice to divide the entire set of samples into two subsets, i.e., the calibration and prediction subsets. This approach enables the behavior of classification models to be assessed by using a different set of samples from the ones used to train or calibrate the model. Thus, the model is calibrated/trained by means of the calibration sample subset. The classification accuracy is assessed by classifying the samples of the prediction set, which are not used in the calibration stage.

**Figure 2:**
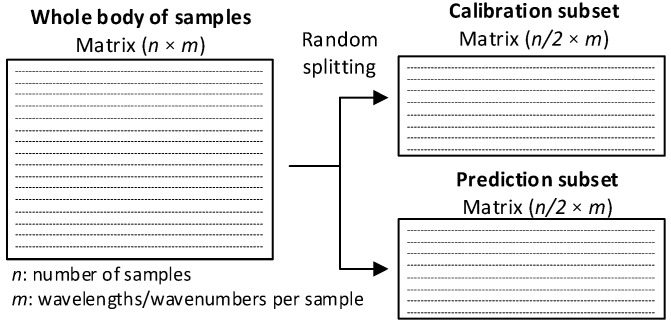
Entire body of samples divided into calibration and prediction sets.

The data analyzed in this work were randomly divided according to the 50–50% proportion, i.e., 50% of the samples were assigned to the calibration set, whereas the remaining 50% of samples were assigned to the prediction set, as shown in Figure 2.

It is noted that the samples of the calibration set were labeled according to the group or class they belonged to. This meant that the class, i.e., its composition, was already known. This is the basis of supervised classification approaches. However, the composition of the prediction set samples was unknown; thus, the classifier had to provide an accurate estimation of their pertinence class, i.e., their composition.

### 4.2. Spectral-Data Preprocessing

To improve the classification results, it is common to preprocess the spectral data prior to the application of the algorithms detailed in Section 4.3 and Section 4.4.

Before any preprocessing, all spectra were transformed into absorbance mode. The first treatment consisted of calculating the first or the second derivatives of the spectral data. The derivatives were calculated by applying the Savitzky–Golay differentiation algorithm, which applies a moving average of five/ten points (first/second derivative, respectively) at each point of the spectrum, thus preventing a reduction in the signal-to-noise ratio due to the derivative operation. Therefore, the spectral data were analyzed in three modes, i.e., raw (without applying any derivative), and first- and second-derivative modes.

Next, to further improve the classification results, spectral data (raw, and first and second derivative) were mean-centered. This operation was performed by subtracting the mean value of each column of the *n* × *m* data matrix to all the *m* variables of each row (every row contained the spectrum of a particular textile sample). The columns of the mean-centered data matrix had zero mean value, but their variances were not modified.

To balance the weights of NIR and MIR spectra for data fusion, the NIR and MIR spectra of all samples were normalized within the 0–1 interval by applying a min–max normalization, *x* = (*x*_1_, *x*_2_,…,*x*_m_), where *x* represents the spectral-data vector of each sample, either NIR or MIR, with *m* components (wavelengths or wavenumbers). The normalized vector, *x_norm_*, was calculated as follows:

(1) $$x_{norm}=\frac{x-\min(x)}{\max(x)-\min(x)}$$

**Figure 3:**
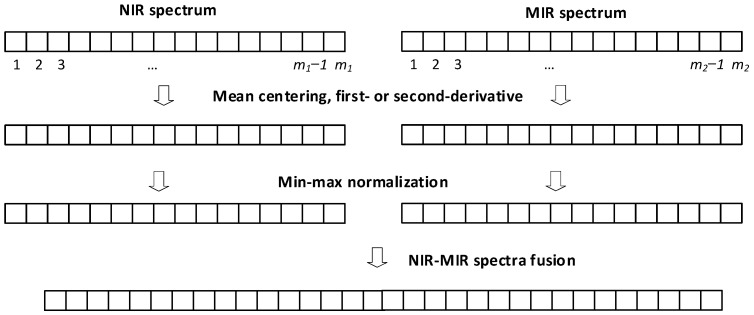
Summary of data-fusion strategy applied in this paper.

Finally, both vectors containing the min–-max normalized NIR an MIR spectra of each sample were merged in only one vector, as shown in Figure 3.

### 4.3. Applied Dimensionality-Reduction Methods

Dimensionality is the number of input variables for a dataset. Dimensionality-reduction methods reduce the number of input variables in a dataset by obtaining a reduced set of new variables, which are combinations of the original variables and essentially include the same information as in the original variables.

Under the experimental conditions of this study, NIR spectrometry provided over two thousand data points per spectrum (textile sample), so it was required to apply dimensionality-reduction techniques, since large numbers of variables tend to cause poor performance of machine learning algorithms.

This paper applied PCA and CVA dimensionality-reduction algorithms, because they condense the analytically relevant data into a reduced set of inferred or latent variables [46,47], which are linear combinations of the original variables. In addition to significantly reducing data dimensionality, these methods also remove experimental noise included in the spectra. Dimensionality-reduction algorithms can be roughly classified as unsupervised or supervised methods. The latter ones usually provide more accurate classification results because they are based on class labels defined by an expert who guides the training process and defines the class labels of the samples of the calibration set [47].

The CVA is a supervised multiclass dimensionality-reduction method that was conceived to maximize the distances among different data classes and simultaneously minimize the distances among samples of the same class for an accurate classification [48]. To this end, a CVA generates non-orthogonal latent variables known as canonical variates or CVs. The number of CVs is equal to the number of classes in the problem minus one. A CVA includes the following steps [49]:

1. A CVA requires a data matrix *X*_(*n*,*m*)_, with *n* being the number of samples and *m* the number of data points or variables in every spectrum;
2. Calculation of dispersion matrix $B_{\left(m,m\right)}=\sum_{i=1}^{c}n_{i}({\bar{x}}_{i}-\bar{x}){\left({\bar{x}}_{i}-\bar{x}\right)}^{T}$, where ${\bar{x}}_{i}=\frac{1}{n_{i}}\sum_{i=1}^{n_{i}}x_{ij}$ with *i* = 1, 2, …, *c* $\bar{x}=\frac{1}{n}\sum_{i=1}^{c}n_{i}{\bar{x}}_{i},\;n=\sum_{i=1}^{c}n_{i}$ and *c* being the number of classes defined in the problem;
3. Calculation of dispersion matrix $W_{\left(m,m\right)}=\sum_{i=1}^{c}\;\sum_{j=1}^{n_{i}}(x_{ij}-{\bar{x}}_{i}){\left(x_{ij}-{\bar{x}}_{i}\right)}^{T}$.
4. Cholesky decomposition of matrix *W*, *W* = *LL^T^*, determining lower triangular matrix *L*;
5. Calculation of matrix *C* = *L*^−^^1^*B*(*L*^−^^1^)*^T^*;
6. Calculation of the *s*-first-normalized eigenvectors (*a_i_*) of *C*, ranked so that the respective eigenvalues are in decreasing order. From them, matrix *A*_(*m*,*s*)_ is obtained, whose columns include normalized eigenvalue. Calculation of the s-first-normalized eigenvectors (*a_i_*) of Cs *a_i_*;
7. Eigenvectors matrix of the original problem is: *V*_(*m*,*s*)_ = (*L*^*T*^)^−^^1^_(*m*,*m*)_ *A*_(*m*,*s*)_;
8. Calculation of the new latent variables: *Y*_(*n*,*s*)_ = *X*_(*n*,*m*)_*V*_(*m*,*s*)_.

A CVA requires datasets with a number of samples greater than the number of original variables. This condition was not fulfilled in this problem, since spectral data have thousands of variables, while the number of samples was limited to a few hundreds. Therefore, it was imperative to reduce the number of variables before applying the CVA algorithm. This reduction was achieved by applying a PCA, one of the most applied unsupervised dimensionality-reduction algorithms [50]; the PCA does not rely on class labels provided by an expert, because the classification training process is not guided. The PCA concentrates the significant chemometric information found in the original set of variables into a reduced set of orthogonal and uncorrelated latent variables called principal components (PCs) [51].

PCs are linear combinations of the original variables, defining orthogonal axes in the space explaining the greatest variance of the entire body of samples. A PCA generates as many PCs as original variables in the problem. The PCs explaining the greatest amount of variance are the ones considered, disregarding the remaining ones. The PCs are ranked in descending order according to the amount of variance they explain, so that the first PC explains the highest percentage of the total variance [52]. The PCA algorithm is as follows [53]:

1. A PCA requires data matrix *X*_(*n*,*m*)_;
2. The first or second derivative of data matrix *X*_(*n*,*m*)_ is calculated, and the resulting matrix is centered if required, thus obtaining the preprocessed data matrix X*_(*n*,*m*)_;
3. Singular-value decomposition of matrix X*_(*n*,*m*)_: *svd*(X*) = *U*_(*n*,*n*)_Σ _(*n*,*m*)_*V*^*T*^_(*m*,*m*)_;
4. Calculation of the new latent variables: *Y*_(*n*,*m*)_ = X*_(*n*,*m*)_*V*_(*m*,*m*)_.

Once sequence PCA + CVA is applied, the dimensionality of the problem is greatly reduced to a number of CVs equal to the number of classes minus one. Next, the classifier can be applied, in this case, *k*NNs, since it is a powerful classifier.

### 4.4. k-Nearest Neighbors (kNNs) for Classification

Once the CVs are obtained, a classifier can be applied. In this paper, the *k*NN classifier was applied because of its wide use, accuracy and simplicity [13,22,48]. The *k*NN output as many normalized values in the [0, 1] interval as the types of textile fibers or classes defined. These output values determined the probability with which the analyzed sample belonged to each class so that the analyzed sample was associated with the class with an output value greater than 0.5. This algorithm is based on the majority-vote rule of the *k*-nearest neighbors of the calibration set, since their pertinence classes were already known. The *k*NN assigned *k* votes to the class of the nearest neighbor, *k*-1 votes to the class of the second nearest neighbor, etc., and one vote to the class of the *k*-th farthest neighbor. The *k*NN also summed the total votes and assigned the analyzed sample to the most voted class.

It is worth noting that all codes were programmed by the authors in the Matlab^®^ environment.

## 5. Data Analyzed

A sequence of three studies was designed. The aim of the first study was to be able to correctly classify 100% pure textile samples (i.e., 100% cotton fibers) among seven different classes (cotton, linen, wool, silk, polyester, polyamide and viscose). In the second study, textile samples were composed of a mixture of viscose and polyester in different percentages. Finally, the third study had the aim of classifying mixtures of cotton/polyester in three classes from high to low cotton percentages.

The NIR and MIR spectra of the samples were registered and then mathematically treated according to the sequence of algorithms described above. The figures below show the absorbance NIR and MIR spectra of the different types of samples.

**Figure 4:**
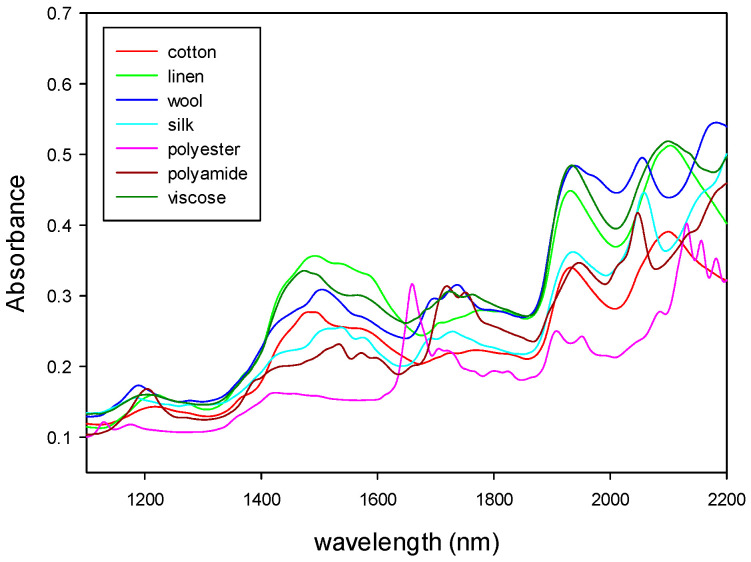
NIR spectra of natural and synthetic fibers.

**Figure 5:**
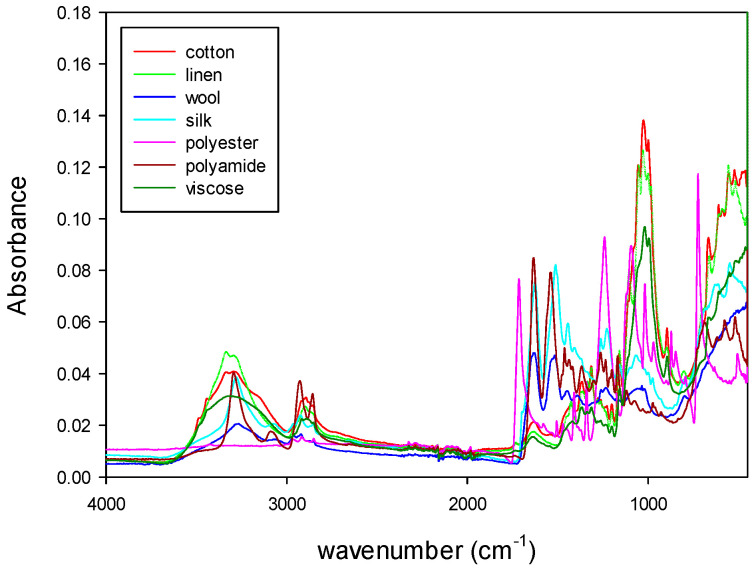
MIR spectra of natural and synthetic fibers.

Figure 4 shows the NIR spectra of natural fibers (cotton, linen, wool and silk) and synthetic fibers (polyester, polyamide and viscose). Figure 5 shows the MIR spectra of the same types of fibers. Both types of spectra show characteristic bands related to the fiber composition. As it can be seen from those figures, MIR spectra (Figure 5) had narrower and more defined pics than NIR, as expected from what was already said about the origin of the absorption bands for each technique.

**Figure 6:**
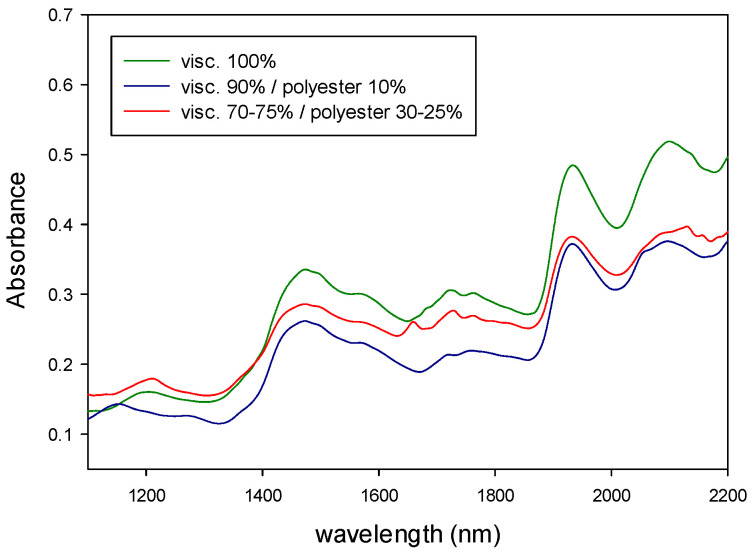
NIR spectra of binary mixtures of viscose/polyester.

**Figure 7:**
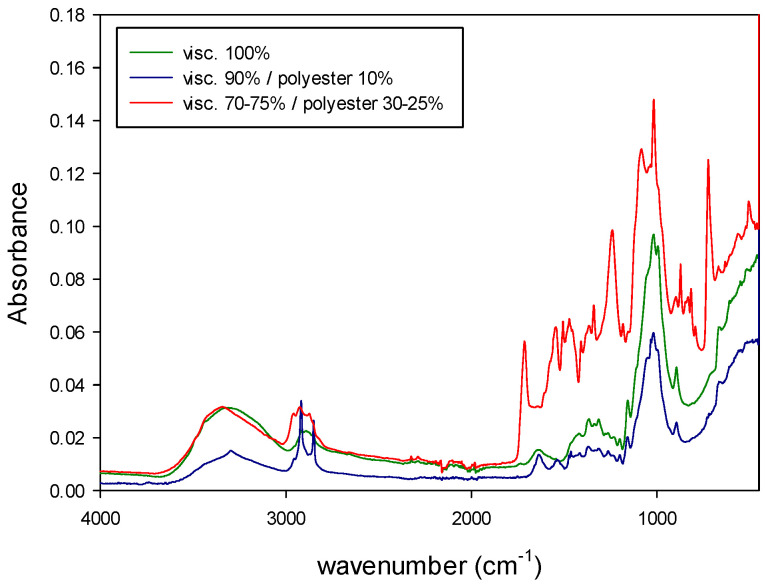
MIR spectra of binary mixtures of viscose/polyester.

**Figure 8:**
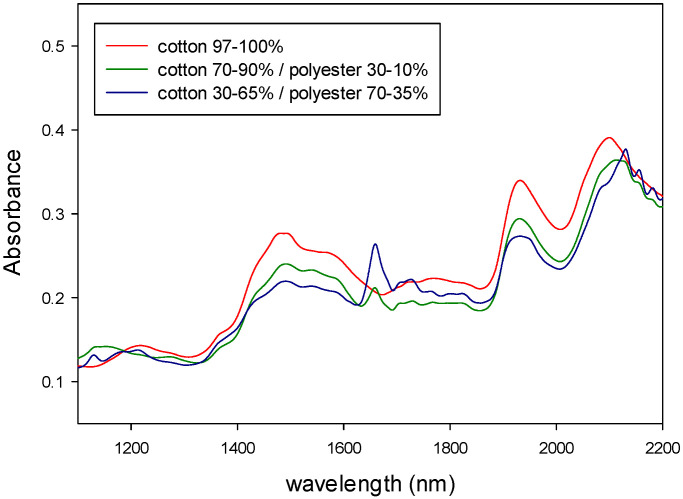
NIR spectra of binary mixtures of cotton/polyester.

**Figure 9:**
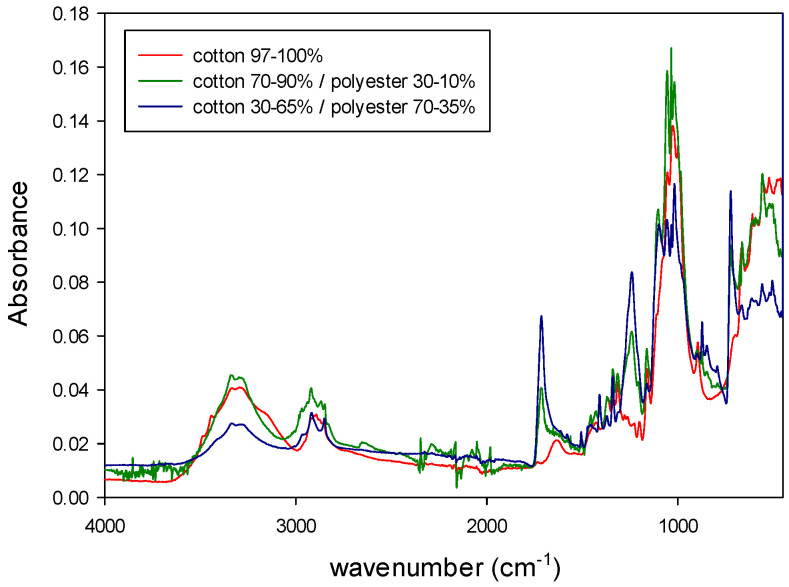
MIR spectra of binary mixtures of cotton/polyester.

In addition, the NIR and MIR spectra of the binary mixtures of fibers are shown in Figure 6 and Figure 7 (viscose/polyester) and Figure 8 and Figure 9 (cotton/polyester), respectively. When carefully observing those figures, the characteristic bands from both components of the blend could be identified.

Figure 4, Figure 5, Figure 6, Figure 7, Figure 8 and Figure 9 show that although some spectra were very similar (i.e., cotton and linen), they presented small differences. As stated above, NIR spectra exhibit wide bands and are thus difficult to interpret. Therefore, the data fusion of NIR and MIR spectra would be useful to obtain more accurate results, especially for complex textile blends where spectra interpretation is even more difficult.

## 6. Experimental Procedures

### 6.1. Study No. 1: Analysis of 210 Pure Fibers

In this study, 210 textile samples were studied. These samples came from pure fibers according to the distribution shown in Table 1.

The samples in Table 1 belong to seven classes (cotton, linen, wool, silk, polyester, polyamide and viscose), so the objective of this study was to assess the classification ability of the analyzed chemometric classification method. To train the mathematical algorithm, the 210 samples were randomly divided into two datasets, i.e., the calibration and prediction sets. Each dataset contained 50% of the textile samples (105 samples, 15 of each of the seven classes), so the calibration and prediction data matrixes contained 105 rows each, whereas the number of columns depended on the study performed (2201 for NIR, 3551 for MIR and 5752 for NIR + MIR).

#### 6.1.1. Study No. 1 with NIR Spectral Data

Table 2 shows the classification errors of the 105 samples of the prediction set using the NIR spectra obtained by applying the PCA + CVA + *k*NN algorithms, with different preprocessing options being applied to the raw spectral data. It is noted that the number of retained PCs was that explaining at least 99.99% of the total variance.

The results presented in Table 2 show the accurate results attained with the proposed classification approach, since in the case of dealing with the second derivative of the NIR spectra, all 105 samples of the prediction set were correctly classified.

It is worth noting that the RRMSE is the relative root mean squared error, which was calculated as:

(2) $$\mathrm{RRMSE}=100\frac{\sqrt{\frac{1}{n}\sum_{i=1}^{n}{\left(y_{meas,i}-y_{pre,i}*\right)}^{2}}}{\frac{1}{n}\sum_{i=1}^{n}y_{meas,i}}$$

where *y*_*meas*,*i*_ is the *i*-th component of a true vector, whereas *y*_*pre*,i_* is the value predicted by the algorithm, which is commonly used to evaluate the performance of mathematical models [54].

Although the initial NIR spectra contained 2201 points each, after the PCA-based dimensionality-reduction stage, only the first PCs explaining 99.99% of the total variance were retained.

#### 6.1.2. Study No. 1 with MIR Spectral Data

Table 3 shows the classification errors of the 105 samples of the prediction set using MIR spectral information.

The results presented in Table 3 show that using the MIR spectra and the PCA + CVA + *k*NN algorithms, this approach correctly classified 104 out of the 105 samples of the prediction set.

#### 6.1.3. Study No. 1 with Combined NIR + MIR Spectral Data

Table 4 shows the classification errors of the 105 samples of the prediction set using combined NIR + MIR spectral information.

The results summarized in Table 4 prove that using the combined NIR + MIR spectra and the PCA + CVA + *k*NN algorithms, it was possible to correctly classify all samples of the prediction set.

### 6.2. Study No. 2: Analysis of Viscose Samples Mixed with Polyester

This second study dealt with 73 textile samples. These samples included pure viscose fibers and viscose mixed with polyester according to the distribution shown in Table 5.

The objective of this study was to correctly classify the three classes defined in Table 5. To achieve this goal, the 73 samples were randomly divided into the calibration and prediction sets, each one containing approximately 50% of the samples as summarized in Table 3.

In this study, the calibration and prediction data matrixes had 37 and 36 rows, respectively, whereas the number of columns depended on the study performed (2201 for NIR, 3551 for MIR and 5752 for NIR + MIR).

#### 6.2.1. Study No. 2 with NIR Spectral Data

Table 6 shows the classification errors of the 36 samples of the prediction set using NIR spectral information.

The results presented in Table 6 show that using the NIR spectra and the PCA + CVA + *k*NN algorithms, it was possible to correctly classify 35 out of the 36 samples of the prediction set.

#### 6.2.2. Study No. 2 with MIR Spectral Data

Table 7 shows the classification errors of the 36 samples of the prediction set using MIR spectral information.

The results in Table 7 show that using the MIR spectra and the PCA + CVA + *k*NN algorithms, it was possible to correctly classify 34 out of the 36 samples of the prediction set.

#### 6.2.3. Study No. 2 with Combined NIR + MIR Spectral Data

Table 8 shows the classification errors of the 36 samples of the prediction set using combined NIR + MIR spectral information.

As seen in Table 8, using the information of the combined NIR + MIR spectra, it was possible to correctly classify all the samples of the prediction set.

### 6.3. Study No. 3. Analysis of Cotton Samples Mixed with Polyester

This third study analyzed 90 samples that came from pure cotton mixed with polyester in different proportions as detailed in Table 9.

The objective of this study was to correctly classify the three classes summarized in Table 9. In this study, the calibration and prediction data matrixes had 45 rows each, whereas the number of columns depended on the study performed (2201 for NIR, 3551 for MIR and 5752 for NIR + MIR).

#### 6.3.1. Study No. 3 with NIR Spectral Data

Table 10 presents the classification errors of the 45 prediction samples obtained by applying the PCA + CVA + *k*NN algorithms to the NIR spectra.

The results in Table 10 show that from the information of the NIR spectra, 40 out of the 45 samples of the prediction set were correctly classified. Some of the five misclassified samples had a special type of finishing, because they were textile samples used for shoes.

#### 6.3.2. Study No. 3 with MIR Spectral Data

Table 11 presents the classification errors of the 45 prediction samples obtained by applying the PCA + CVA + *k*NN algorithms to the MIR spectra.

The results presented in Table 11 show that using the MIR spectra and the PCA + CVA + *k*NN algorithms, it was possible to correctly classify 40 out of the 45 samples of the prediction set.

#### 6.3.3. Study No. 3 with Combined NIR + MIR Spectral Data

Table 12 shows the classification errors of the 45 samples of the prediction set using combined NIR + MIR spectral information.

The results presented in Table 12 show that using the combined NIR + MIR spectra and the PCA + CVA + *k*NN algorithms, it was possible to correctly classify 43 out of the 45 samples of the prediction set.

### 6.4. Summary of Studies No. 1, No. 2 and No. 3

This section summarizes the results attained in the three studies performed in this paper for a fair comparison of the attained results. Table 13 summarizes the mean results obtained in each study with the three spectral datasets.

The results presented in Table 13 clearly show that the best results were always attained when combining the spectral information provided by the NIR and MIR techniques.

The sources of errors were most probably the number and nature of the samples. In general, the more samples used in the calibration are, the better the prediction is. Nevertheless, really good prediction results were obtained in this case, at least with pure samples (study No. 1). Samples with a mixture of fibers were more difficult to classify, especially when classifying very wide groups (the composition of the samples belonging to the same group widely varied around 20–35%) and groups close to each other (around 5% difference in composition), as in the case of study No. 3. In this case, a greater number of samples in the calibration set would be advisable to obtain a better prediction rate.

## 7. Conclusions

The main conclusion of the present work is that the data fusion of NIR and MIR spectra provides more accurate classification results than NIR or MIR separately, especially for the classification of similar samples (i.e., binary mixtures where the classes are very close to one another).

The classification of pure fibers into seven types (cotton, linen, wool, silk, polyester, polyamide and viscose) took place with 100% accuracy (no misclassified samples) using either NIR or data-fusion spectra as input information. On the other hand, with binary mixtures of fibers, data fusion always provided the best classification results (2 out of 45 misclassified samples, with 5% errors, were obtained in the worst case, cotton/polyester). The three groups defined for the cotton/polyester blends (cotton/polyester) were very broad and close, i.e., cotton ≥ 97%, cotton at 70–90% and cotton at 30–65%. In contrast, the viscose/polyester mixtures were three groups that were narrower and more separated from each other, i.e., viscose at 100%, viscose at 90% and viscose at 70–75%.

Data fusion is a very promising alternative when the classes that need to be accurately distinguished have narrow borders between them. This is expected to be the case in textile-waste classification for recycling purposes.

These are excellent results for a possible application of the proposed method in textile-waste classification to ensure the quality and composition of the recycled materials.

## Figures and Tables

**Table 1:** Textile Samples Analyzed in Study No. 1.

Type	Composition	Number of Samples (Calibration/Prediction)
Natural fiber	Cotton, 100%	30 (15/15)
Natural fiber	Linen, 100%	30 (15/15)
Natural fiber	Wool, 100%	30 (15/15)
Natural fiber	Silk, 100%	30 (15/15)
Synthetic fiber	Polyester, 100%	30 (15/15)
Synthetic fiber	Polyamide, 100%	30 (15/15)
Artificial fiber	Viscose, 100%	30 (15/15)

**Table 2:** Study No. 1 with NIR Spectral Data. Prediction-Set Classification Errors (105 samples).

Processing Type		PCA + CVA + *k*NN			
		*k* = 3	*k* = 4	*k* = 5	*k* = 6
Mean centering	Errors	3/105	2/105	2/105	2/105
	RRMSE	0.0636	0.0648	0.0650	0.0657
1st derivative + mean centering	Errors	1/105	1/105	1/105	1/105
	RRMSE	0.0497	0.0497	0.0497	0.0497
2nd derivative + mean centering	Errors	0/105	0/105	0/105	0/105
	RRMSE	0.0000	0.0000	0.0000	0.0024

**Table 3:** Study No. 1 with MIR Spectral Data. Prediction-Set Classification Errors (105 Samples).

Processing Type		PCA + CVA + *k*NN			
		*k* = 3	*k* = 4	*k* = 5	*k* = 6
Mean centering	Errors	1/105	1/105	1/105	1/105
	RRMSE	0.0510	0.0516	0.0517	0.0517
1st derivative + mean centering	Errors	2/105	2/105	2/105	2/105
	RRMSE	0.0703	0.0703	0.0680	0.0670
2nd derivative + mean centering	Errors	1/105	1/105	1/105	1/105
	RRMSE	0.0497	0.0499	0.0501	0.0502

**Table 4:** Study No. 1 with combined NIR + MIR spectral data. Prediction-set classification errors (105 samples).

Processing Type		PCA + CVA + *k*NN			
		*k* = 3	*k* = 4	*k* = 5	*k* = 6
Mean centering	Errors	1/105	1/105	1/105	1/105
	RRMSE	0.0497	0.0497	0.0497	0.0497
1st derivative + mean centering	Errors	0/105	0/105	0/105	0/105
	RRMSE	0.0185	0.0211	0.0212	0.0204
2nd derivative + mean centering	Errors	0/105	0/105	0/105	0/105
	RRMSE	0.0000	0.0000	0.0000	0.0024

**Table 5:** Textile Samples Analyzed in Study No. 2.

Composition	Number of Samples (Calibration/Prediction)
Viscose, 100%	26 (13/13)
Viscose, 90%/Polyester, 10%	26 (13/13)
Viscose, 70–75%/Polyester, 30–25%	21 (11/10)

**Table 6:** Study No. 2 with NIR spectral data. Prediction-set classification errors (36 samples).

Processing Type		PCA + CVA + *k*NN			
		*k* = 3	*k* = 4	*k* = 5	*k* = 6
Mean centering	Errors	3/36	2/36	2/36	2/36
	RRMSE	0.5233	0.4676	0.4314	0.4085
1st derivative + mean centering	Errors	2/36	2/36	2/36	2/36
	RRMSE	0.5346	0.5346	0.5346	0.5346
2nd derivative + mean centering	Errors	1/36	1/36	1/36	1/36
	RRMSE	0.1890	0.2268	0.2520	0.2700

**Table 7:** Study No. 2 with MIR spectral data. Prediction-set classification errors (36 samples).

Processing Type		PCA + CVA + *k*NN			
		*k* = 3	*k* = 4	*k* = 5	*k* = 6
Mean centering	Errors	2/36	2/36	2/36	2/36
	RRMSE	0.5346	0.5306	0.5298	0.5312
1st derivative + mean centering	Errors	6/36	6/36	6/36	6/36
	RRMSE	0.9259	0.9259	0.9259	0.9259
2nd derivative + mean centering	Errors	4/36	4/36	4/36	4/36
	RRMSE	0.7560	0.7560	0.7560	0.7560

**Table 8:** Study No. 2 with Combined NIR + MIR Spectral Data. Prediction-Set Classification Errors (36 Samples).

Processing Type		PCA + CVA + *k*NN			
		*k* = 3	*k* = 4	*k* = 5	*k* = 6
Mean centering	Errors	0/36	0/36	0/36	0/36
	RRMSE	0.0000	0.0000	0.0000	0.0000
1st derivative + mean centering	Errors	0/36	0/36	0/36	0/36
	RRMSE	0.0000	0.0000	0.0000	0.0000
2nd derivative + mean centering	Errors	1/36	1/36	1/36	1/36
	RRMSE	0.3780	0.3780	0.3780	0.3780

**Table 9:** Textile samples analyzed in study No. 3.

Composition	Number of Samples (Calibration/Prediction)
Cotton ≥ 97%	30 (15/15)
Cotton, 70–90%/Polyester, 30–10%	30 (15/15)
Cotton, 30–65%/Polyester, 70–35%	30 (15/15)

**Table 10:** Study No. 3 with NIR Spectral Data. Prediction-Set Classification Errors (45 Samples).

Processing Type		PCA + CVA + *k*NN			
		*k* = 3	*k* = 4	*k* = 5	*k* = 6
Mean centering	Errors	17/45	12/45	10/45	10/45
	RRMSE	0.8226	0.7973	0.7848	0.7775
1st derivative + mean centering	Errors	9/45	8/45	7/45	7/45
	RRMSE	0.7199	0.7156	0.7113	0.7099
2nd derivative + mean centering	Errors	5/45	5/45	5/45	5/45
	RRMSE	0.6048	0.6048	0.6048	0.6048

**Table 11:** Study No. 3 with MIR Spectral Data. Prediction-Set Classification Errors (45 Samples).

Processing Type		PCA + CVA + *k*NN			
		*k* = 3	*k* = 4	*k* = 5	*k* = 6
Mean centering	Errors	8/45	8/45	8/45	8/45
	RRMSE	0.7311	0.7166	0.7049	0.6991
1st derivative + mean centering	Errors	5/45	5/45	5/45	5/45
	RRMSE	0.5860	0.5738	0.5659	0.5605
2nd derivative + mean centering	Errors	5/45	5/45	5/45	5/45
	RRMSE	0.6065	0.6072	0.6072	0.6070

**Table 12:** Study No. 3 with Combined NIR + MIR Spectral Data. Prediction-Set Classification Errors (45 Samples).

Processing Type		PCA + CVA + *k*NN			
		*k* = 3	*k* = 4	*k* = 5	*k* = 6
Mean centering	Errors	3/45	3/45	3/45	3/45
	RRMSE	0.4685	0.4685	0.4590	0.4499
1st derivative + mean centering	Errors	2/45	2/45	2/45	2/45
	RRMSE	0.3825	0.3825	0.3825	0.3825
2nd derivative + mean centering	Errors	3/45	3/45	3/45	3/45
	RRMSE	0.4685	0.4685	0.4685	0.4685

**Table 13:** Summary of the Results Attained in the Three Studies from the NIR, MIR and NIR + MIR Spectral Information.

		Spectral Information		
Study		NIR	MIR	NIR + MIR
Study #1	Errors	1.08/105	1.33/105	0.33/105
	RRMSE	0.0382	0.0568	0.0235
Study #2	Errors	1.75/36	4.00/36	0.33/36
	RRMSE	0.4089	0.7378	0.1260
Study #3	Errors	8.33/45	6.00/45	2.67/45
	RRMSE	0.7048	0.6305	0.4375

## Data Availability

Not applicable.
